# Human, canine and feline strongyloidiasis: beyond *Strongyloides stercoralis*

**DOI:** 10.1017/S0031182025100644

**Published:** 2025-09

**Authors:** Huan Zhao, Constantin Constantinoiu, Richard Bradbury

**Affiliations:** 1 School of Public Health and Tropical Medicine, College of Medicine and Dentistry, James Cook University, Townsville, QLD, Australia; 2 School of Veterinary Science, College of Science and Engineering, James Cook University, Townsville, QLD, Australia

**Keywords:** epidemiology, Pets, *Strongyloides*, *Strongyloides fuelleborni*, strongyloidiasis, zoonoses

## Abstract

*Strongyloides stercoralis* has historically dominated research and control efforts for strongyloidiasis in both medical and veterinary fields. This has obscured the significance of other *Strongyloides* species infecting humans and their closest companions, dogs (*Canis lupus familiaris*) and cats (*Felis catus*). This review synthesized clinical and epidemiologic evidence on these neglected agents of human and companion animal strongyloidiasis and outlined priorities for future research. Our aim is to raise awareness of these understudied species and promote research to clarify their medical and veterinary public health significance. Targeted species-specific surveillance using molecular-genomic and advanced morphological tools is essential to uncover the true burden of these infections and inform strategies for their control and eventual elimination.

## Graphical abstract

**Figure:**
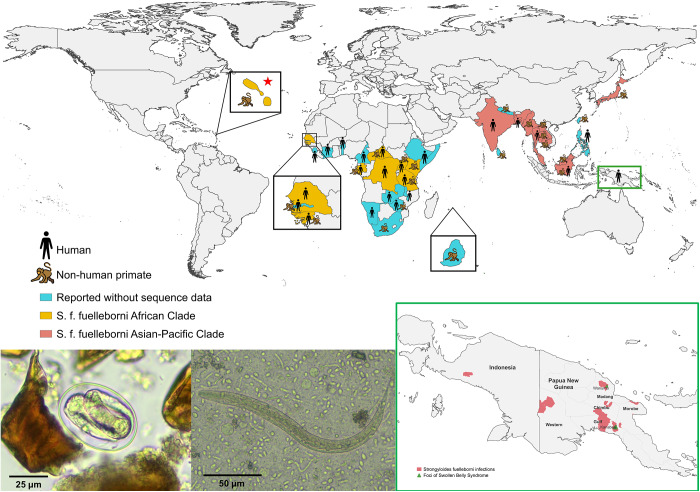

## Introduction

Soil-transmitted nematodes of the genus *Strongyloides* infect a wide range of mammals, including humans (*Homo sapiens*) and their closest companion animals, dogs (*Canis lupus familiaris*) and cats (*Felis catus*) (Al-Jawabreh et al, 2024). *Strongyloides stercoralis* is the primary agent of human and canine strongyloidiasis and has historically been the focus of research and control efforts in both medical and veterinary contexts (Buonfrate et al, 2023; Al-Jawabreh et al, 2024). *Strongyloides fuelleborni* subsp. *fuelleborni*, historically considered a rare zoonosis acquired from non-human primates (NHPs) (Potters et al, 2020), has been identified at unexpectedly high prevalence in human populations in parts of Asia and Africa (Pampiglione and Ricciardi, 1972b; de Ree et al, 2024). Emerging reports of this species from Papua New Guinea (PNG) (Zhao et al, 2025), along with mounting evidence of human-to-human transmission (Hira and Patel, 1980; Hasegawa et al, 2016), suggest that *S. f. fuelleborni* is likely underreported globally (Buonfrate et al, 2022). A third agent of human strongyloidiasis, *Strongyloides fuelleborni* subsp. *kellyi*, is endemic to the island of New Guinea where it has been associated with a severe, often fatal protein-losing enteropathy in infants, known as ‘swollen belly syndrome’ (SBS) (Ashford et al, 1992; Bradbury, 2021). Recent molecular evidence suggests that *S. f. kellyi* may be synonymous with the Asian-Pacific clade of *S. f. fuelleborni* (Zhao et al, 2025).

*Strongyloides* infections in dogs and cats remain relatively underexplored. In addition to *S. stercoralis*, 3 other *Strongyloides* species have been identified in cats (Chandler AC, 1925; Rogers, 1939; Price and Dikmans, 1941); however, their veterinary clinical significance and public health relevance remain poorly understood (Zhao and Bradbury, 2024). There is ongoing debate about the existence of a dog-infecting *Strongyloides* sp. that is taxonomically distinct from *S. stercoralis* (Jaleta et al, 2017; Barratt et al, 2019; Bradbury et al, 2021; Bradbury and Streit, 2024), referred to by early researchers as ‘*Strongyloides canis*’ (Brumpt, 1922). This discussion has gained renewed relevance with the recent identification of a dog-specific *S. stercoralis* lineage (*cox*1 lineage B) (Jaleta et al, 2017; Nagayasu et al, 2017; Barratt et al, 2019). Furthermore, cryptic genospecies of dog-infecting *Strongyloides* have been identified in remote Australian communities (Beknazarova et al, 2019).

Diagnosis of strongyloidiasis in both medical and veterinary contexts has traditionally relied on morphological identification of larvae (as in *S. stercoralis* and *Strongyloides felis*) or embryonated eggs (as in *S. fuelleborni* and *Strongyloides planiceps*) in faeces, a method with limited sensitivity due to intermittent larval shedding and low parasite burden (Buonfrate et al, 2022). Parasitic females are small and often embedded deep within the intestinal mucosa, making detection difficult by necropsy (Speare, 1986; Buonfrate et al, 2022). Molecular diagnostics such as real-time PCR (qPCR), though increasingly available, generally do not provide species-level resolution (Buonfrate et al, 2022, 2023). Accurate identification of non-*S. stercoralis* infections in humans and companion animals often requires advanced morphological analysis of cultured free-living adult stages or molecular genotyping, both of which demand substantial expertise and time. This may lead to underdiagnosis or misattribution to *S. stercoralis* due to its well-known medical and veterinary impact. A recently developed duplex qPCR assay can differentiate between *S. stercoralis* and *S. f. fuelleborni*, but its sensitivity is markedly lower than that of the most widely used genus-level assay (Cunningham et al, 2025), although higher than that of faecal conventional PCR-based genotyping approaches for *S. fuelleborni* identification (Barratt et al, 2019).

The longstanding research focus on *S. stercoralis* has eclipsed the study of other *Strongyloides* spp. that are potentially relevant to human and animal health. This review summarises evidence on non-*S. stercoralis* aetiological agents of strongyloidiasis in humans, dogs and cats. Our aim is to raise awareness of these neglected and underexplored species and to promote research that will clarify their medical and veterinary public health significance.

## Human strongyloidiasis

### Strongyloides fuelleborni fuelleborni

*Strongyloides fuelleborni fuelleborni* is a common parasite of NHPs (Al-Jawabreh et al, 2024). Human infection with *S. f. fuelleborni* was first reported in 1932 in Zimbabwe, Southern Africa (Blackie, 1932). Wallace et al (1948) documented the first human case from Asia in 1948. Between 1968 and 1972, Pampiglione and Ricciardi (1972b) conducted a survey of *S. f. fuelleborni* infections in 4577 individuals across 13 nations, spanning the breadth of West to East Africa. Infection was detected in 13% (606/4577) of individuals, with prevalence by locality ranging from 0% to 78% (Pampiglione and Ricciardi, 1972b). Prevalence was higher in children compared to adults, and infection was endemic in tropical rainforest localities, but only sporadic in Savannah environments (Pampiglione and Ricciardi, 1972b). Subsequent examination of diagnostic specimens in two Zambian communities found *S. f. fuelleborni* prevalence of 10% (13/131) (Hira and Patel, 1977) and 31% (138/448) (Hira and Patel, 1980), respectively.

**Figure 1.**
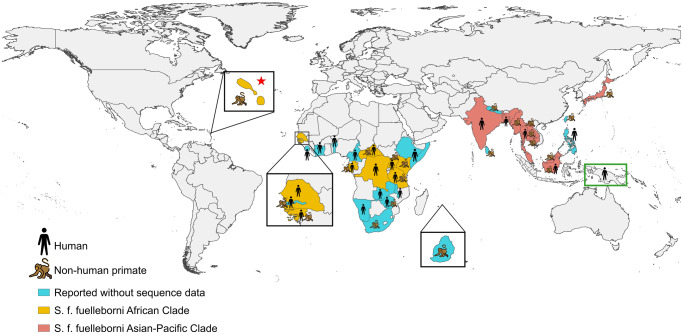
Global distribution of *Strongyloides fuelleborni fuelleborni* in humans and non-human primates (NHPs). African and Asian-Pacific clades of *S. f. fuelleborni*, inferred from available genbank sequences of *cox1, 18S rrna* HVR-IV and mitochondrial genome regions, are shown in yellow and pink, respectively. *Strongyloides f. fuelleborni* infecting St. Kitts vervet monkeys (*Chlorocebus aethiops sabaeus*), indicated by a red asterisk, were introduced from Africa in the 17th century (RICHINS et al., 2023). The distribution of *S. fuelleborni* on the Island of New Guinea, marked with a green box, remains unresolved (see Figure 2).

Since then, no active surveillance for *S. f. fuelleborni* in humans have been undertaken. However, over the past decade, human infections have been increasingly reported in sub-Saharan Africa (Hasegawa et al, 2010, 2016; Barratt et al, 2019; Potters et al, 2020), Southeast Asia (Labes et al, 2011; Thanchomnang et al, 2017; Janwan et al, 2020), and South Asia (Barratt et al, 2019; de Ree et al, 2024). While most reports involve isolated cases, a genotyping survey in Bangladesh identified *S. f. fuelleborni* infections in 3% (4/134) of people from four communities (de Ree et al, 2024). Most recently, *S. f. fuelleborni* was molecularly detected in 37% (7/19) of infant stool samples collected from the Eastern Highlands Province of PNG (Zhao et al, 2025). These findings suggest that *S. f. fuelleborni* may be far more prevalent and geographically widespread in human populations than currently recognized (Figure 1). Microscopy may misidentify *S. f. fuelleborni* eggs as morphologically similar embryonated hookworm eggs and hatched larvae are indistinguishable from those of *S. stercoralis* (Speare, 1986). Additionally, the lack of targeted control strategies for strongyloidiasis globally may contribute to sustained transmission (Lo et al, 2025). In regions where mass drug administration (MDA) with ivermectin, the most effective chemical against *S. stercoralis*, has been implemented for the control of onchocerciasis or lymphatic filariasis, *S. f. fuelleborni* prevalence may have been incidentally reduced, as has been observed for *S. stercoralis* (Stroffolini et al, 2023). Nonetheless, without targeted surveillance, the true distribution and burden of *S. f. fuelleborni* remain unknown and likely underestimated.

There is limited understanding of the clinical presentation of *S. f. fuelleborni* infections. Most clinical insights come from a historical human experimental infection, which showed a broad spectrum of clinical signs, some resembling those seen in *S. stercoralis* infection (Pampiglione and Ricciardi, 1972a). These included localized dermatologic manifestations (such as urticaria and/or ground itch) at the onset, followed by transient, non-productive cough and gastrointestinal symptoms (epigastric burning, abdominal pain and diarrhea) in later stages. Marked eosinophilia (up to 48%) was observed 3–4 weeks post-infection (Pampiglione and Ricciardi, 1972a). It should be noted that this study used a human strain of *S. f. fuelleborni*, which had previously been inoculated into the same participant at a lower dose in a preliminary experiment (Pampiglione and Ricciardi, 1972a). Therefore, the observed symptoms may have been influenced by prior sensitization and/or immune response mounted to *S. f. fuelleborni* antigens. As this species is passed in the environment as eggs, it has been assumed that an internal autoinfective cycle does not occur (Centers for Disease Control and Prevention, 2019); however, this assumption has not been experimentally confirmed. It remains possible that small numbers of eggs could hatch in the gut or perianal folds and develop into filariform larvae and reinfect the host, although existing epidemiologic data do not support this hypothesis (Pampiglione and Ricciardi, 1972b).

The transmission patterns of *S. f. fuelleborni* in human populations are similarly not fully understood. Both NHP-to-human (Sandground, 1925; Blackie, 1932; Freedman, 1991) and human-to-human (Pampiglione and Ricciardi, 1972a) transmissions have been experimentally demonstrated. Human infections have historically been considered a zoonosis from NHPs. This is supported by molecular evidence showing identical or closely clustered genotypes in worms isolated from humans and NHPs living in close proximity in Tanzania (Hasegawa et al, 2010), the Democratic Republic of the Congo (Potters et al, 2020) and Thailand (Thanchomnang et al, 2017; Janwan et al, 2020). However, growing evidence suggests that exclusively interhuman transmission is also possible. Hira and Patel (1980) found high prevalence in people from urban and peri-urban communities in Zambia, where contact with NHPs was unlikely. Likewise, Hasegawa et al (2016) observed marked genetic divergence at the *cox1* and *18S rRNA* HVR-IV loci in worms from humans (*n* = 7) and NHPs (*n* = 18) cohabiting the Dzanga-Sangha Protected Area of the Central African Republic. These findings, together with emerging evidence of human *S. f. fuelleborni* infections in PNG where NHPs are absent (Zhao et al, 2025), suggest that this parasite has adapted to sustained human-to-human transmission in some parts of the world. Brown and Girardeau (1977) noted the transmammary passage of *Strongyloides* infective filariform larvae (iL3), suspected to be *S. f. fuelleborni*, from one of 26 African nursing mothers to her infant. This finding requires further confirmation, as only a single larva was morphometrically characterised and found to be relatively small (340 µm) (Brown and Girardeau, 1977), so the possibility of this being an autoinfective larva of *S. stercoralis* cannot be excluded based on the size and morphology alone.

### Strongyloides fuelleborni kellyi

*Strongyloides fuelleborni kellyi* was first reported by Allen Kelly in 1971 during a stool microscopy survey in western PNG (Kelly and Voge, 1973). Due to its morphological similarity in adult stages to *S. fuelleborni* von Linstow, 1905, but the absence of a NHP reservoir in New Guinea, it was designated a subspecies of *S. fuelleborni* and named *S. f. kellyi* (Viney et al, 1991). A separate isoenzyme electrophoretic analysis grouped African and most PNG *S. fuelleborni* isolates together; however, 4 PNG isolates clustered with *Strongyloides ransomi* from local pigs (Viney and Ashford, 1990). Viney and Ashford (1990) speculated that these findings might represent artifact from participants submitting pig faeces in substitution for human samples. Subsequently, phylogenetic analysis of a 330 bp *18S rRNA* fragment from a formalin-fixed human isolate from PNG by Dorris et al (2002) placed the parasite within a clade containing *Strongyloides venezuelensus* and *S. ransomi*, but separate from *S. f. fuelleborni*. This placement was supported by a recent genotyping analysis of *18S rRNA* HVR-IV (252–259 bp) and HVR-I (432 bp) loci. Most importantly, this study demonstrated the co-occurrence, in one of 19 infants, of the genospecies identified by Dorris et al (2002) alongside the Asian clade of *S. f. fuelleborni* in PNG (Zhao et al, 2025).

**Figure 2.**
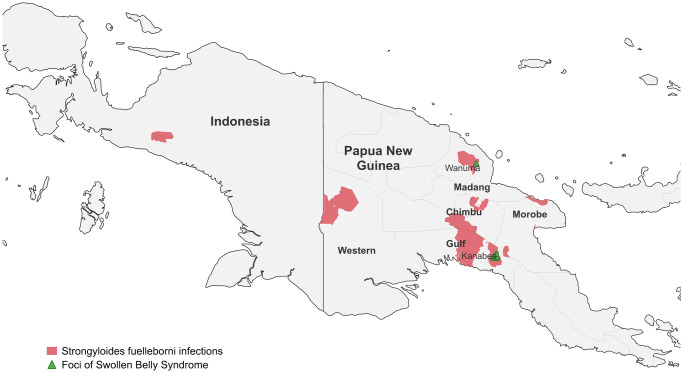
Human infections with *S. fuelleborni* in New Guinea. The data presented may also include infections caused by an undescribed *strongyloides* sp. genetically distinct from *S. fuelleborni*, as molecularly demonstrated by Dorris et al. (2002) and Zhao et al. (2025). Sites of confirmed SBS outbreaks are indicated by green triangleS. Sporadic SBS cases have also been reported elsewhere in Papua New Guinea (data not shown).

The co-endemicity of two genetically distinct non-*S. stercoralis* human-infecting *Strongyloides* nematodes in PNG necessitates a reassessment of historical data previously attributed to *S. f. kellyi* (Figure 2). The parasite described by Viney et al (1991) and designated *S. f. kellyi* may represent the Asian-Pacific clade of *S. f. fuelleborni*, whereas the genospecies identified by Dorris et al (2002) and Zhao et al (2025) may represent an undescribed *Strongyloides* sp., potentially of animal origin. As there exists no morphological studies of *S. f. fuelleborni* from Asia, further comparative morphologic and genomic analyses of adult isolates from Africa, Asia and New Guinea are needed to resolve this taxonomic confusion.

Historical epidemiologic and clinical data on *S. f. kellyi*, generated prior to the molecular era, also warrant re-evaluation (Speare, 1986; Viney et al, 1991). Faecal microscopy surveys conducted between 1976 and 1997 in PNG reported *S. f. kellyi* prevalence ranging from 20% to 93% in children and 5% to 20% in adults (Ashford and Babona, 1980; Ashford et al, 1979; Barnish and Ashford, 1989a, b; Barnish and Harari, 1989; Kelly et al, 1976; King and Mascie-Taylor, 2004; Shield et al, 1987; Shield and Kow, 2013). Infections were detected in children as young as 18 days, with prevalence peaking between 30 and 60 months of age and declining after 5 years (Ashford et al, 1992; Barnish and Ashford, 1989a, 1989b). Faecal egg count could reach up to 100 000 epg in late infancy (Barnish and Ashford, 1989a). Human infections have also been reported in Deiyai Regency, in the Indonesian province of Central Papua (Muller et al, 1987). No faecal surveys for *Strongyloides* have been conducted in New Guinea since 1997. However, two community-based serosurveys reported *Strongyloides* seroprevalence of 22.5% (27/120) (Scott et al, 2022) in Western Province by a *S. stercoralis* L3 crude antigen ELISA, and 68% (192/283) in Madang Province by a dual NIE and SsIR recombinant antigen ELISA (Tobon Ramos et al, 2025). In the absence of cross-reactivity data for these assays, the proportion of seropositivity attributable to *S. f. kellyi* remains unknown.

Uncertainty also surrounds the transmission pattern of *S. f. kellyi*. Ashford et al (1992) postulated that heavy infections in infancy may result from repeated exposure to iL3 within soiled straw bags (bilums) used to carry infants. Transmammary transmission has also been suspected; however, a survey of breastmilk from lactating women in a PNG community did not detect any larvae, although the infection status of the mothers and their infants was not assessed (Barnish and Ashford, 1989a). No zoonotic reservoir has been identified, despite investigations into local pigs, chickens and dogs (Kelly and Voge, 1973; Viney and Ashford, 1990).

In PNG, *S. f. kellyi* infection has been uniquely associated with an acute, fatal infantile protein losing enteropathy known as SBS (Ashford et al, 1992). Clinical features include hypoproteinemia, abdominal distension, respiratory distress, eosinophilia, diarrhea and peripheral oedema (Ashford et al, 1992). A remarkably similar syndrome, characterized by villus atrophy, malabsorption, hypoproteinaemia and sudden death, has been described in newborn piglets infected with *S. ransomi* (Enigk and Dey-Hazra, 1975). Between 1974 and 1983, SBS cases were predominantly reported from 2 regions of PNG, Kanabea in Gulf Province and Wanuma in Madang Province (Ashford et al, 1979; Vince et al, 2005). Elsewhere in the country, SBS was rare and sporadic (Ashford et al, 1992). In Kanabea, approximately 96 cases were recorded, accounting for 8% of infantile mortality (Ashford et al, 1979). Affected infants often passed large numbers of *Strongyloides* eggs (Ashford et al, 1979), although some high-intensity infections resulted in malnutrition without SBS (Barnish and Harari, 1989; King and Mascie-Taylor, 2004). A co-factor in the pathogenesis of SBS has been suggested (Ashford et al, 1992). Given renewed evidence on the co-endemicity of *S. f. fuelleborni* (potentially synonymous with *S. f. kellyi*) and an undescribed genospecies closely related to *S. ransomi* in PNG (Zhao et al, 2025), it remains plausible that the latter may be the true aetiologic agent of SBS. This hypothesis is supported by clinical parallels to *S. ransomi*-induced disease in piglets. Future investigations into strongyloidiasis and SBS cases in PNG should employ species-specific molecular diagnostics and careful morphological characterization to accurately identify the causative agent and establish the epidemiologic link.

## Canine strongyloidiasis

*Strongyloides stercoralis* is currently recognized as the only *Strongyloides* sp. naturally infecting dogs (Speare, 1986; Thamsborg et al, 2017). In immunocompetent dogs, infection is often asymptomatic or subclinical, though clinical signs such as diarrhoea, bronchopneumonia, emaciation and lethargy may occur, particularly in juvenile animals (Paradies et al, 2017; Thamsborg et al, 2017; Basso et al, 2019). Severe systemic disease has been documented in puppies and immunocompromised adult dogs, often with fatal outcomes (Paradies et al, 2017; Bourgoin et al, 2018; Alves et al, 2021; Unterköfler et al, 2022; Nosková et al, 2024).

Natural infection of dogs with *S. planiceps* has been suggested in two studies (Arizono, 1976; Horie et al, 1980). Horie et al (1980) experimentally infected cats with a *Strongyloides* sp. isolated from dogs and subsequently detected embryonated eggs in feline faeces, leading to the suspicion that the isolate was *S. planiceps*. Arizono (1976) described a strain identified as *S. planiceps,* reportedly isolated from a dog in Japan and serially maintained in puppies. However, neither study provided detailed morphological confirmation of the species, so it remains unclear whether dogs are indeed natural hosts of *S. planiceps*. Patent experimental infections of dogs with *S. f. fuelleborni* (Sandground, 1925) and *Strongyloides procyonis* (Little, 1966) have been documented, but these are not the intended focus of this review.

### Does ‘Strongyloides canis’ exist?

Friedrich Fülleborn first reported natural *S. stercoralis* infection in dogs in 1911 (Fulleborn, 1914). In the years that followed, debate emerged over the taxonomic identity of this canine parasite. Despite being morphologically indistinguishable from human strains, Brumpt (1922) postulated that the dog-derived *S. stercoralis* may represent a separate species. This was based on observed differences in geographical distribution and some life cycle characteristics between human and canine strains, along with unfruitful experimental attempts to establish persistent infections in dogs using human-derived worms (Braun, 1899; Brumpt, 1922; Sandground, 1925; Fülleborn, 1927). He proposed naming the canine parasite ‘*Strongyloides canis*’ (Brumpt, 1922). However, this designation was largely disregarded in subsequent decades primarily due to the lack of morphological justification (Speare, 1986).

This debate has gained renewed attention in the molecular era. Population genetics studies based on partial regions of *cox1, 18S rRNA* and whole-genome data support the existence of a dog-only lineage of *S. stercoralis* (*cox*1 lineage B), alongside a dog-cat-primate shared lineage (*cox1* lineage A) (Jaleta et al, 2017; Nagayasu et al, 2017; Barratt et al, 2019). It has been hypothesized that human-infecting *S. stercoralis* may be a host-adapted variant of an ancestral canine parasite (Nagayasu et al, 2017; Liu et al, 2025). Several researchers suggested that this ancestral dog-restricted population, potentially *S. stercoralis cox*1 lineage B, may represent ‘*S. canis*’ as proposed by Brumpt (Jaleta et al, 2017; Barratt et al, 2019; Barratt and Sapp, 2020; Bradbury et al, 2021).

However, recent genomic analyses have revealed a more complex landscape. Liu et al (2025) demonstrated that *S. stercoralis* infecting humans and dogs in Asia consisted of 2 largely genomically separable but not reproductively isolated populations. This suggests that human and dog lineages may not be taxonomically distinct, as evidenced by occasional introgression between the two (Liu et al, 2025). Similarly, de Ree et al (2024) identified *S. stercoralis cox*1 lineage B in a Bangladesh human. These findings do not rule out the existence of ‘*S. canis*’, but caution against oversimplifying genotyping results based on single genes or short gene regions. Further whole-genome analysis of worms from a broader geographical range, coupled with detailed morphological analysis, is necessary to confirm or refute the hypothesis of *‘S. canis*’ as a distinct species or a subspecies of *S. stercoralis*.

### Canine cryptic strongyloides species

In a faecal metabarcoding survey conducted in remote northern Australia, Beknazarova et al (2019) identified a *Strongyloides* sp. in 2 dog samples that clustered basally to all known *S. stercoralis* isolates on a 217 bp *cox1* region. One of these dogs also harboured unique *18S rRNA* HVR-I and HVR-IV haplotypes (genotype VIII/F) (Beknazarova et al, 2019). These findings suggest the possible existence of a novel, undescribed species, or a genetically distinct strain or subspecies of *S. stercoralis,* in Australian dogs. However, considering that coprophagy is very common in dogs, the possibility that the detected *Strongyloides* DNA originated from ingested material rather than true infection cannot be ruled out. Further morphological and long-read genetic sequencing analyses are needed to clarify its identity.

## Feline strongyloidiasis

### Strongyloides felis

*Strongyloides felis* was first described by Chandler (1925) in cats from India in 1925. Since then, only 3 studies have reported this species (Speare and Tinsley, 1986, 1987; Jitsamai, 2019). In two faecal (Baermann) surveys conducted in North Queensland, Australia, *S. felis* infection was morphologically confirmed in 41% (83/203) (Speare and Tinsley, 1986) and 33.5% (169/504) (Speare and Tinsley, 1987) of shelter cats, respectively. A third study in Thailand identified rhabditiform *Strongyloides* larvae in 1.7% (14/835) of feline faecal samples by PBS–ethyl acetate centrifugal sedimentation microscopy; adults cultured from 6 of these were morphologically identified as *S. felis* (Jitsamai, 2019). However, this morphological identification is dubious, as it described a hexagonal stoma in the free-living female whereas this feature is characteristic of the parasitic female (Speare, 1986). Larvae in the remaining positive samples were not identified to species level (Jitsamai, 2019) and could represent *S. felis* or other feline *Strongyloides* spp.

No other reports of *S. felis* are available, and its true prevalence and distribution remain obscure. Given its substantial morphological similarity to *S. stercoralis* (Speare, 1986), *S. felis* is likely underdiagnosed. Both species characteristically shed larvae, rather than eggs, in faeces (Speare, 1986; Speare and Tinsley, 1986). Differentiation of these two species requires detailed morphological analysis of stomal and tail shape in the parasitic female and vulval morphology in the free-living female. *Strongyloides felis* is distinguished by a rectangular stoma shape and a more finely tapered tail in the parasitic female, and by the presence of post-vulval narrowing and posterior vulval rotation in the free-living female (Speare, 1986). Morphometrics alone cannot reliably differentiate most *Strongyloides* spp., including *S. stercoralis* and *S. felis* (Speare, 1986). Accurate identification currently relies on a very advanced level of parasitological expertise, skills which have been largely lost from the parasitology community (Bradbury et al, 2022). Therefore, the development of species-specific molecular tools is urgently needed to support future studies.

The clinical picture of *S. felis* infection in cats is not fully clear. This parasite is considered moderately pathogenic in cats, based on observations from both natural and experimental infections (Speare, 1986; Speare and Tinsley, 1986). Pathological changes include adenomatous metaplasia of the glandular epithelium in the intestinal crypts, where parasitic females reside. Larval migration may cause pulmonary inflammation, with frequent interstitial changes or focal haemorrhage. Watery diarrhoea has been noted in some high-burden infections, though it is not a consistent feature (Speare and Tinsley, 1986).

*Strongyloides felis* appears to infect adult cats more commonly than kittens. In the study by Speare and Tinsley (1986), prevalence of *S. felis* was found to be 56% (77/138) in adult cats compared to only 9% (6/65) in kittens. The infection tends to be long-lasting; experimentally infected cats maintained patent infections for over a year (Speare and Tinsley, 1986). These epidemiological features resemble those of *S. stercoralis* in humans and dogs, indicating the likelihood of autoinfection and potential lifelong infections (Buonfrate et al, 2023; Al-Jawabreh et al, 2024). Transmission of *S. felis* in cats is thought to occur predominantly via skin penetration by iL3 from the environment. In a survey of 65 kittens, no infection was found in those under 3 months of age, suggesting that transmammary transmission is unlikely (Speare and Tinsley, 1986).

### Strongyloides tumefaciens

Price and Dikmans (1941) first described *S. tumefaciens* in cats from the USA in 1941. During necropsy, multiple tumour-like lesions, some of which were haemorrhagic, were observed in the colonic wall of infected cats. Adult worms were found within the nodules but not in the colonic lumen. These pathological features were considered unique among feline *Strongyloides* spp. Based on these, and the apparent larger body length of *S. tumefaciens* (5000 µm) compared to other known feline *Strongyloides* spp. (<3330 µm) in the parasitic female, a new species was designated (Price and Dikmans, 1941).

Remarkably, another necropsy survey of cats from St. Kitts (Wulcan et al, 2019) observed similar colonic nodules in cats infected by *S. stercoralis*. The recovered adult worms were morphologically indistinguishable from *S. tumefaciens* as described by Price and Dikmans (1941); however, phylogenetic analysis of a 522 bp region of *cox1* placed them within the *S. stercoralis* lineage A (Wulcan et al, 2019). These findings challenged the taxonomic validity of *S. tumefaciens*.

Since its original description, *S. tumefaciens* has been reported in cats from the USA (Malone et al, 1977; Lindsay et al, 1987), Brazil (Moura et al, 2016) and India (Dubey and Â, 1964). In all instances, species identification was based solely on the presence of colonic nodules, which Wulcan et al (2019) indicated to be unreliable.

### Strongyloides planiceps

*Strongyloides planiceps* was initially reported by R.T. Leiper in rusty tiger cats (*Prionailurus rubiginosus*) from Malaysia and later described in domestic cats by Rogers (1939). A distinguishing feature of *S. planiceps* is that larvated eggs, rather than rhabditiform larvae, are shed in faeces (Rogers, 1939; Speare, 1986). Morphologically, the parasitic female of *S. planiceps* has spiralled ovaries and a bluntly rounded tail, contrasting with the straight ovaries and narrowly tapered tail of *S. stercoralis* and *S. felis* (Rogers, 1939; Speare, 1986). Unlike *S. stercoralis*, the life cycle of *S. planiceps* involves multiple free-living generations, up to 9, as demonstrated experimentally (Yamada et al, 1991).

*Strongyloides planiceps* is believed to primarily infect wild felids and only sporadically occur in domestic cats (Horie et al, 1981; Fukase et al, 1983; El-Seify et al, 2017). Reports of *S. planiceps* have almost exclusively come from Japan (Arizono, 1976; Horie et al, 1980, 1981; Fukase et al, 1983, 1985; Sato et al, 2006). Although numerous feline surveys from other countries have reported egg-shedding *Strongyloides* spp. in cat faeces, none identified the parasites to the species level, so it is unknown whether they represent *S. planiceps* (Abbas et al, 2022; Adams et al, 2008; Adhikari et al, 2023; Borkataki et al, 2013; de Sousa^1^ et al. 2015; Heidt et al, 1988; Monteiro et al, 2016; Nyambura Njuguna et al, 2017; Solórzano-García et al, 2017; Susilowati, 1985). One study from Egypt found *S. planiceps* in one of 170 cat faecal samples, but the method for confirming species was not reported (El-Seify et al, 2017). It is likely that this helminth is significantly underreported in cats globally.

### Feline cryptic Strongyloides species

Two studies reported molecular evidence of a *Strongyloides* sp. in cats (Jitsamai, 2019; Ko et al, 2020). Ko et al (2020) analysed partial *cox*1 and protein-coding mitochondrial genome sequences of 70 *Strongyloides* isolates from 19 cats in Myanmar and found that they formed a sister taxon to *S. stercoralis*. A similar finding was reported by Jitsamai (2019) based on a 708 bp region of *18S rRNA*. It has been suggested that the *Strongyloides* detected in both studies may represent *S. felis*, but without morphological confirmation, this remains speculative.

## Conclusions and future directions

Beyond *S. stercoralis,* multiple other *Strongyloides* spp. infect humans and companion animals. Yet these remain grossly understudied due to diagnostic limitations, scarce morphological and molecular data and a historical research focus on *S. stercoralis*. This review synthesized evidence on these neglected nematodes, aiming to raise awareness and encourage further research to clarify their significance in medical and veterinary public health. Below, we highlight key research gaps and propose priorities for future research:

a) Major gaps remain in the global burden and epidemiology of *S. f. fuelleborni* infections in humans. Mounting evidence of interhuman transmission suggests that this parasite may be more widely disseminated via human migration than currently understood. In parallel, the translocation of infected NHP may serve as a mobile zoonotic reservoir. Invasive NHP species have been reported in several Pacific regions, including New Guinea (Kemp and Carter, 2022). Transmission among introduced or imported NHP, as demonstrated by Richins et al (2023) and Juhasz et al (2023), may pose a risk to local humans. Large-scale, species-specific surveillance is needed to define the true prevalence, geographic range, and public health relevance of *S. f. fuelleborni*.

b) The taxonomic, epidemiologic, and clinical landscape of *S. f. kellyi* infection requires re-evaluation using species-discriminative molecular diagnostics. Comparative morphological analysis of adult isolates from Asia, Africa and New Guinea, combined with mitochondrial and whole-genome sequencing, is needed to determine whether *S. f. kellyi* is truly synonymous with the Asian-Pacific clade of *S. f. fuelleborni*. Molecular taxonomy based on *18S rRNA* loci suggests the existence of an undescribed human-infecting *Strongyloides* sp. in PNG (Dorris et al, 2002; Zhao et al, 2025). This hypothesis requires validation through detailed morphological characterization of worms of all life stages combined with genomic analysis. Renewed surveillance for strongyloidiasis and SBS in New Guinea is needed to clarify the causative species and their respective public health significance.

c) The potential existence of a dog-specific population of *S. stercoralis*, historically referred to as ‘*S. canis*’, remains unresolved. Whole-genome analysis of isolates from humans and dogs across diverse and sympatric settings will be essential to delineate host specificity and transmission dynamics. Additionally, cryptic *Strongyloides* genospecies identified in Australian dogs require further research to determine whether they represent true infections or reflect transient DNA passage or sequencing artefacts.

d) *Strongyloides* spp. infecting cats remain overall poorly understood. Species-specific surveillance globally using molecular and morphological tools, combined with veterinary clinical data, is needed to clarify their prevalence, distribution, veterinary impact and potential zoonotic relevance.

## References

[ref1] Abbas I, Al-Araby M, Elmishmishy B and El-Alfy E-S (2022) Gastrointestinal parasites of cats in Egypt: High prevalence high zoonotic risk. *BMC Veterinary Research* 18(1): 420. 36447265 10.1186/s12917-022-03520-0 PMC9706847

[ref2] Adams P, Elliot A, Algar D and Brazell R (2008) Gastrointestinal parasites of feral cats from Christmas Island. *Australian Veterinary Journal* 86(1‐2), 60–63. 18271830 10.1111/j.1751-0813.2007.00246.x

[ref3] Adhikari RB, Dhakal MA, Ale PB, Regmi GR and Ghimire TR (2023) Survey on the prevalence of intestinal parasites in domestic cats (*Felis catus* Linnaeus, 1758) in central Nepal. *Veterinary Medicine and Science* 9(2): 559–571. 36346533 10.1002/vms3.999 PMC10029910

[ref4] Al-Jawabreh R, Anderson R, Atkinson LE, Bickford-Smith J, Bradbury RS, Breloer M, Bryant AS, Buonfrate D, Cadd LC, Crooks B, Deiana M, Grant W, Hallem E, Hedtke SM, Hunt V, Khieu V, Kikuchi T, Kounosu A, Lastik D, van Lieshout L, Liu Y, McSorley HJ, McVeigh P, Mousley A, Murcott B, Nevin WD, Nosková E, Pomari E, Reynolds K, Ross K, Streit A, Suleiman M, Tiberti N and Viney M (2024) *Strongyloides* questions-a research agenda for the future. *Philosophical Transactions of the Royal Society of London B: Biological Sciences* 379(1894): 20230004. doi:10.1098/rstb.2023.0004 38008122 PMC10676812

[ref5] Alves RC, Soares YG, dos S, Pinheiro JK, Brito Junior JRC, Silva RAF, Firmino M, de O, and Dantas AFM (2021) Strongyloidiasis in a puppy in Northeastern Brazil. *Acta Scientiae Veterinariae* 49. doi:10.22456/1679-9216.113326

[ref6] Arizono N (1976) Studies on the free-living generations of *Strongyloides planiceps* Rogers, 1943. I. Effects of quantity of food and population density on the developmental types. *Japanese Journal of Parasitology* 25(4): 274–282.

[ref7] Ashford R and Babona D (1980) The parasites of the purari people of gulf province, papua new guinea. *Papua and New Guinea Medical Journal* 23(4): 165–168. 6937025

[ref8] Ashford RW, Barnish G and Viney ME (1992) *Strongyloides fuelleborni kellyi*: Infection and disease in Papua New Guinea. *Parasitology Today* 8(9): 314–318. doi:10.1016/0169-4758(92)90106-C 15463651

[ref9] Ashford RW, Vince JD, Gratten MA and Bana-Koiri J (1979) *Strongyloides* infection in a mid-mountain Papua New Guinea community: Results of an epidemiological survey. *Papua New Guinea Medical Journal* 22(2): 128–135. 298723

[ref10] Barnish G and Ashford R (1989a) *Strongylaides cf. fuelleborni* and hookworm in Papua New Guinea: Patterns of infection within the community. *Transactions of the Royal Society of Tropical Medicine and Hygiene* 83(5): 684–688. 2617632 10.1016/0035-9203(89)90398-2

[ref11] Barnish G and Ashford R (1989b) *Strongyloides cf fuelleborni* in Papua New Guinea: Epidemiology in an isolated community, and results of an intervention study. *Annals of Tropical Medicine and Parasitology* 83(5): 499–506. 2619364 10.1080/00034983.1989.11812378

[ref12] Barnish G and Harari M (1989) Possible effects of *Strongyloides fuelleborni*-like infections on children in the Karimui area of Simbu Province. *Papua and New Guinea Medical Journal* 32(1): 51–54. 2750322

[ref13] Barratt JL, Lane M, Talundzic E, Richins T, Robertson G, Formenti F, Pritt B, Verocai G, Nascimento de Souza J and Mato Soares N (2019) A global genotyping survey of *Strongyloides stercoralis* and *Strongyloides fuelleborni* using deep amplicon sequencing. *PLoS Neglected Tropical Diseases* 13(9): e0007609. 31525192 10.1371/journal.pntd.0007609 PMC6762204

[ref14] Barratt JL and Sapp SG (2020) Machine learning-based analyses support the existence of species complexes for *Strongyloides fuelleborni* and *Strongyloides stercoralis*. *Parasitology* 147(11): 1184–1195. 32539880 10.1017/S0031182020000979 PMC7443747

[ref15] Basso W, Grandt L-M, Magnenat A-L, Gottstein B and Campos M (2019) Strongyloides stercoralis infection in imported and local dogs in Switzerland: From clinics to molecular genetics. *Parasitology Research* 118(1): 255–266. 30552576 10.1007/s00436-018-6173-3

[ref16] Beknazarova M, Barratt JL, Bradbury RS, Lane M, Whiley H and Ross K (2019) Detection of classic and cryptic *Strongyloides* genotypes by deep amplicon sequencing: A preliminary survey of dog and human specimens collected from remote Australian communities. *PLoS Neglected Tropical Diseases* 13(8): e0007241. 31430282 10.1371/journal.pntd.0007241 PMC6716672

[ref17] Blackie W (1932) *A Helminthological Survey of Southern Rhodesia*. Keppel Street, Gower Street, W.C.I. London:School of Hygiene & Tropical Medicine.

[ref18] Borkataki S, Katoch R, Goswami P, Godara R, Khajuria J, Yadav A and Kaur R (2013) Prevalence of parasitic infections of stray cats in Jammu, India. *Sokoto Journal of Veterinary Sciences* 11(1): 1–6.

[ref19] Bourgoin G, Jacquet‐Viallet P and Zenner L (2018) Fatal strongyloidiasis in a puppy from France. *Veterinary Record Case Reports* 6(2): e000415.

[ref20] Bradbury RS (2021) *Strongyloides fuelleborni kellyi* in New Guinea: Neglected, ignored and unexplored. *Microbiology Australia* 42(4): 169–172.

[ref21] Bradbury RS, Pafčo B, Nosková E and Hasegawa H (2021) *Strongyloides* genotyping: A review of methods and application in public health and population genetics. *International Journal for Parasitology* 51(13-14): 1153–1166. 34757088 10.1016/j.ijpara.2021.10.001

[ref22] Bradbury RS, Sapp SG, Potters I, Mathison BA, Frean J, Mewara A, Sheorey H, Tamarozzi F, Couturier MR and Chiodini P (2022) Where have all the diagnostic morphological parasitologists gone?. *Journal of Clinical Microbiology* 60(11): e00986–00922. 36314793 10.1128/jcm.00986-22 PMC9667774

[ref23] Bradbury RS and Streit A (2024) Is strongyloidiasis a zoonosis from dogs?. *Philosophical Transactions of the Royal Society B* 379(1894): 20220445. 10.1098/rstb.2022.0445 PMC10676807 38008118

[ref24] Braun M (1899) Bemerkungen fiber d. sporad. Fall von A. intestinalis in Ostpreussen. *Centrabl Bakt Parasit* 26, 612–615.

[ref25] Brown RC and Girardeau H (1977) Transmammary passage of *Strongyloides* sp. larvae in the human host. *The American Journal of Tropical Medicine and Hygiene* 26(2): 215–219. 848643 10.4269/ajtmh.1977.26.215

[ref26] Brumpt E (1922) *Strongyloides Stercoralis (Bavay, 1877). Précis de Parasitologie*, 3ème edition. Paris: *Mason et Cie*, 691–697.

[ref27] Buonfrate D, Bradbury RS, Watts MR and Bisoffi Z (2023) Human strongyloidiasis: Complexities and pathways forward. *Clinical Microbiology Reviews* 36(4): e00033–00023. doi:10.1128/cmr.00033-23 37937980 PMC10732074

[ref28] Buonfrate D, Tamarozzi F, Paradies P, Watts MR, Bradbury RS and Bisoffi Z (2022) The diagnosis of human and companion animal *Strongyloides stercoralis* infection: Challenges and solutions. A scoping review. *Advances in Parasitology* 118, 1–84. 36088083 10.1016/bs.apar.2022.07.001

[ref29] Centers for Disease Control and Prevention (2019) Strongyloidiasis. Available at https://www.cdc.gov/dpdx/strongyloidiasis/index.html (accessed 16 June 2025).

[ref30] Chandler AC (1925) The species of *Strongyloides* (Nematoda). *Parasitology* 17, 426–433. 10.1017/S0031182000004856

[ref31] Cunningham L, Nevin W, Verweij J, Buonfrate D, Scarso S, Khieu V, O’Ferrall A, Rollason S and Stothard R (2025) Improving molecular epidemiological surveillance of strongyloidiasis upon differentiation of *Strongyloides fuelleborni fuelleborni* from *Strongyloides stercoralis*. *Journal of Infectious Diseases*. doi:10.1093/infdis/jiaf237 PMC12308652 40423557

[ref32] de Ree V, Nath TC, Barua P, Harbecke D, Lee D, Rödelsperger C and Streit A (2024) Genomic analysis of *Strongyloides stercoralis* and *Strongyloides fuelleborni* in Bangladesh. *PLoS Neglected Trop Dis* 18(9): e0012440. doi:10.1371/journal.pntd.0012440 PMC11407627 39226300

[ref33] de Sousa¹ TN, de Sousa¹ ACB, and dos Santos Sousa¹ DG and Freire SM (2015) Ocorrência de parasitos gastrintestinais de gatos (Felis catus) que frequentam a Universidade Estadual do Piauí, Campus Torquato Neto, Teresina (PI) Occurrence of gastrointestinal parasites of cats (Felis catus) attending the State University of Piauí. *Ca. Pubvet* 8, 2806–2887.

[ref34] Dorris M, Viney ME and Blaxter ML (2002) Molecular phylogenetic analysis of the genus *Strongyloides* and related nematodes. *International Journal for Parasitology* 32(12); 1507–1517. 12392916 10.1016/s0020-7519(02)00156-x

[ref35] Dubey J and Â P (1964) Helminthic lesions encountered in the alimentary canal of the Indian wild cat (*Felis chaus*.). *Agra University Journal of Research* 13, 169–184.

[ref36] El-Seify MA, Aggour MG, Sultan K and Marey NM (2017) Gastrointestinal helminths of stray cats in Alexandria, Egypt: A fecal examination survey study. *Veterinary Parasitology: Regional Studies and Reports* 8, 104–106. 31014624 10.1016/j.vprsr.2017.03.003

[ref37] Enigk K and Dey-Hazra A (1975) Intestinal plasma and blood loss in piglets infected with *Strongyloides ransomi*. *Veterinary Parasitology* 1(1): 69–75. 877430

[ref38] Freedman DO (1991) Experimental infection of human subjects with *Strongyloides* species. *Reviews of Infectious Diseases* 13(6): 1221–1226. 1775856 10.1093/clinids/13.6.1221

[ref39] Fukase T, Chinone S and Itagaki H (1983) *Strongyloides planiceps* (Nematoda; Strongyloididae) from cats in Kanagawa Prefecture, Japan. *Journal of the Japan Veterinary Medical Association* 36, 589–592. 10.1292/jvms1939.47.627 4046267

[ref40] Fukase T, Chinone S and Itagaki H (1985) *Strongyloides planiceps* (Nematoda; Strongyloididae) in some wild carnivores. *Nihon Juigaku Zasshi* 47(4): 627–632. doi:10.1292/jvms1939.47.627 4046267

[ref41] Fulleborn F (1914) Untersuchungen uber den Infektionsweg bei *Strongyloides* und Ankylostomum und die Biologie dieser Parasiten. *Archiv für Schiffs- und Tropen-Hygiene* 18, 26–80.

[ref42] Fülleborn F (1927) *Über Das Verhalten der Larven von Strongyloides Stercoralis, Hakenwürmern Und Ascaris Lumbricoides Im Körper Des Wirtes Und Ein Versuch, Es Biologisch Zu Deuten*. Leipzig: Johann Ambrosius Barth.

[ref43] Hasegawa H, Kalousova B, McLennan MR, Modry D, Profousova-Psenkova I, Shutt-Phillips KA, Todd A, Huffman MA and Petrzelkova KJ (2016) *Strongyloides* infections of humans and great apes in Dzanga-Sangha Protected Areas, Central African Republic and in degraded forest fragments in Bulindi, Uganda. *Parasitology International* 65(5 Pt A), 367–370. doi:10.1016/j.parint.2016.05.004 27180094

[ref44] Hasegawa H, Sato H, Fujita S, Nguema PPM, Nobusue K, Miyagi K, Kooriyama T, Takenoshita Y, Noda S and Sato A (2010) Molecular identification of the causative agent of human strongyloidiasis acquired in Tanzania: Dispersal and diversity of *Strongyloides* spp. and their hosts. *Parasitology International* 59(3): 407–413. 20621633 10.1016/j.parint.2010.05.007

[ref45] Heidt GA, Rucker RA, Kennedy ML and Baeyens ME (1988) Hematology, intestinal parasites, and selected disease antibodies from a population of bobcats (*Felis rufus*) in central Arkansas. *Journal of Wildlife Diseases* 24(1): 180–183. 3352091 10.7589/0090-3558-24.1.180

[ref46] Hira P and Patel B (1977) *Strongyloides fülleborni* infections in man in Zambia. *The American Journal of Tropical Medicine and Hygiene* 26(4): 640–643. 889005 10.4269/ajtmh.1977.26.640

[ref47] Hira PR and Patel BG (1980) Human strongyloidiasis due to the primate species *Strongyloides fülleborni*. *Tropical Geographic Medicine Association* 32(1): 23–29. 7394891

[ref48] Horie M, Noda R, Noda S and Onishi T (1980) Studies on *Strongyloides* sp. isolated from a dog. II. Experimental infections in cats. *Japanese Journal of Parasitology* 29(1): 45–54.

[ref49] Horie M, Noda S, Noda R and Higashino J (1981) Studies on *Strongyloides* sp. isolated from a cat and raccoon dog. *Japanese Journal of Parasitology* 30, 215–223.

[ref50] Jaleta TG, Zhou S, Bemm FM, Schär F, Khieu V, Muth S, Odermatt P, Lok JB and Streit A (2017) Different but overlapping populations of *Strongyloides stercoralis* in dogs and humans—Dogs as a possible source for zoonotic strongyloidiasis. *PLoS Neglected Tropical Diseases* 11(8): e0005752. 28793306 10.1371/journal.pntd.0005752 PMC5565190

[ref51] Janwan P, Rodpai R, Intapan PM, Sanpool O, Tourtip S, Maleewong W and Thanchomnang T (2020) Possible transmission of *Strongyloides fuelleborni* between working Southern pig-tailed macaques (Macaca nemestrina) and their owners in Southern Thailand: Molecular identification and diversity. *Infection Genetics & Evolution* 85, 104516. 10.1016/j.meegid.2020.104516 32860989

[ref52] Jitsamai W (2019) Prevalence of enteric helminths and protozoa and identification of hookworm, threadworm and giardia spp. In *Cats in Bangkok and Vicinity, Thailand*. Chulalongkorn University Theses and Dissertations (Chula ETD), 8914. https://digital.car.chula.ac.th/chulaetd/8914.

[ref53] Juhasz A, Spiers E, Tinsley E, Chapman E, Shaw W, Head M, Cunningham LJ, Archer J, Jones S and Haines LR (2023) Gastrointestinal parasites in captive olive baboons in a UK safari park. *Parasitology* 150(12): 1096–1104. 37655745 10.1017/S0031182023000823 PMC10801365

[ref54] Kelly A, Little M and Voge M (1976) *Strongyloides fulleborni*-like infections in man in Papua New Guinea. *The American Journal of Tropical Medicine and Hygiene* 25(5): 694–699. 961992 10.4269/ajtmh.1976.25.694

[ref55] Kelly A and Voge M (1973) Report of a nematode found in humans at Kiunga, Western District. *Papua New Guinea Medical Journal* 16, 59.

[ref56] Kemp N and Carter S (2022) Macaca fascicularis. crab-eating macaque). Available at (accessed). 10.1079/cabicompendium.76108

[ref57] King SE and Mascie-Taylor C (2004) *Strongyloides fuelleborni kellyi* and other intestinal helminths in children from Papua New Guinea: Associations with nutritional status and socioeconomic factors. *Papua New Guinea Medical Journal* 47(3/4): 181–191. 16862942

[ref58] Ko PP, Suzuki K, Canales-Ramos M, MPPTH H, Htike WW, Yoshida A, Montes M, Morishita K, Gotuzzo E and Maruyama H (2020) Phylogenetic relationships of *Strongyloides* species in carnivore hosts. *Parasitology International* 78, 102151. 32502520 10.1016/j.parint.2020.102151

[ref59] Labes E, Wijayanti N, Deplazes P and Mathis A (2011) Genetic characterization of *Strongyloides* spp. from captive, semi-captive and wild Bornean orangutans (*Pongo pygmaeus*) in Central and East Kalimantan, Borneo, Indonesia. *Parasitology* 138(11), 1417–1422. 21838961 10.1017/S0031182011001284

[ref60] Lindsay D, Blagburn B, Stuart B and Gosser H (1987) *Strongyloides tumefaciens* infection in a cat. *Companion Animal Practice* 1(1), 12–13.

[ref61] Little MD (1966) Seven new species of Strongyloides (Nematoda) from Louisiana. *The Journal of Parasitology* 52(1), 85–97. 5932110

[ref62] Liu Y, Ahmr S, Sripa B, Tangkawattana S, Khieu V, Nevin W, Paterson S and Viney M (2025) A population genetic analysis of the nematode *Strongyloides stercoralis* in Asia shows that human infection is not a zoonosis from dogs. *Proceedings of the National Academy of Sciences* 122(29), e2424630122. doi:10.1073/pnas.2424630122 PMC12304889 40663613

[ref63] Lo NC, Addiss DG, Buonfrate D, Amor A, Anegagrie M, Bisoffi Z, Bradbury RS, Keiser J, Kepha S and Khieu V (2025) Review of the WHO guideline on preventive chemotherapy for public health control of strongyloidiasis. *The Lancet Infectious Diseases* 25(3), e146–e152. 39481419 10.1016/S1473-3099(24)00595-4 PMC11871984

[ref64] Malone J, Butterfield A, Williams J, Stuart B and Travasos H (1977) *Strongyloides tumefaciens* in cats. *Journal of the American Veterinary Medical Association* 171(3), 278–280. 893214

[ref65] Monteiro MFM, Ramos RAN, Calado AMC, Lima VFS, ICdN R, Tenório RFL, MAdG F and Alves LC (2016) Gastrointestinal parasites of cats in Brazil: Frequency and zoonotic risk. *Revista Brasileira de Parasitologia Veterinária* 25, 254–257. 27096530 10.1590/S1984-29612016019

[ref66] Moura MAO, Jorge EM, KKGd N, Riet-Correa G, Abel I, Cavalcante GG, CAd O and Bezerra PS (2016) Colonic epithelial nodular hyperplasia associated with strongyloidiasis in cats in the Amazon region, Pará State, Brazil. *Ciência Rural* 47(1), e20160328.

[ref67] Muller R, Lillywhite J, Bending J and Catford J (1987) Human cysticercosis and intestinal parasitism amongst the Ekari people of Irian Jaya. *The Journal of Tropical Medicine and Hygiene* 90(6), 291–296. 3430662

[ref68] Nagayasu E, Mppthh A, Hortiwakul T, Hino A, Tanaka T, Higashiarakawa M, Olia A, Taniguchi T, Win SMT and Ohashi I (2017) A possible origin population of pathogenic intestinal nematodes, *Strongyloides stercoralis*, unveiled by molecular phylogeny. *Scientific Reports* 7(1), 4844. 28687738 10.1038/s41598-017-05049-x PMC5501853

[ref69] Nosková E, Svobodová V, Hypská V, Cerezo-Echevarria A, Kurucová T, Ilík V, Modrý D and Pafčo B (2024) High-throughput sequencing of Strongyloides stercoralis–a fatal disseminated infection in a dog. *Parasitology* 151(6), 587–593. 38800868 10.1017/S0031182024000568 PMC11427980

[ref70] Nyambura Njuguna A, Kagira JM, Muturi Karanja S, Ngotho M, Mutharia L and Wangari Maina N (2017) Prevalence of *Toxoplasma gondii* and other gastrointestinal parasites in domestic cats from households in Thika region, Kenya. *BioMed Research International* 2017(1), 7615810. 28691033 10.1155/2017/7615810 PMC5485282

[ref71] Pampiglione S and Ricciardi M (1972a) Experimental infestation with human strain *Strongyloides fülleborni* in man. *The Lancet* 299(7752), 663–665. 10.1016/s0140-6736(72)90464-3 4125165

[ref72] Pampiglione S and Ricciardi M (1972b) Presence of *Strongyloides fülleborni* in man in tropical Africa. Further epidemiological studies. Human experimental infection. *Bull Soc Path Exot Filiales* 65(1), 112–119. 4677863

[ref73] Paradies P, Iarussi F, Sasanelli M, Capogna A, Lia RP, Zucca D, Greco B, Cantacessi C and Otranto D (2017) Occurrence of strongyloidiasis in privately owned and sheltered dogs: Clinical presentation and treatment outcome. *Parasites and Vectors* 10(1), 345. 28728589 10.1186/s13071-017-2275-5 PMC5520385

[ref74] Potters I, Micalessi I, Van Esbroeck M, Gils S and Theunissen C (2020) A rare case of imported *Strongyloides fuelleborni* infection in a Belgian student. *Clinical Infection in Practice* 7, 100031.

[ref75] Price E and Dikmans G (1941) Adenomatous tumors in the large intestine of cats caused by Strongyloides tumefaciens n. sp. *Proceedings of the Helminthological Society of Washington, D.C* 8.

[ref76] Richins T, Sapp SG, Ketzis JK, Willingham AL, Mukaratirwa S, Qvarnstrom Y and Barratt JL (2023) Genetic characterization of *Strongyloides fuelleborni* infecting free-roaming African vervets (*Chlorocebus aethiops sabaeus*) on the Caribbean island of St. Kitts. *International Journal for Parasitology: Parasites and Wildlife* 20, 153–161. 36860205 10.1016/j.ijppaw.2023.02.003 PMC9969202

[ref77] Rogers W (1939) A new species of *Strongyloides* from the cat. *Journal of Helminthology* 17(4), 229–238.

[ref78] Sandground J (1925) Speciation and specificity in the nematode genus *Strongyloides*. *The Journal of Parasitology* 12(2), 59–80.

[ref79] Sato H, Suzuki K, Osanai A, Kamiya H and Furuoka H (2006) Identification and characterization of the threadworm, *Strongyloides procyonis*, from feral raccoons (*Procyon lotor*) in Japan. *The Journal of Parasitology* 92(1), 63–68. 16629317 10.1645/GE-623R.1

[ref80] Scott J, Emeto TI, Melrose W, Warner J and Rush C (2022) Seroepidemiology of *Strongyloides* spp. Infection in Balimo, Western Province, Papua New Guinea. *The American Journal of Tropical Medicine and Hygiene* 108(2), 346. 36572010 10.4269/ajtmh.22-0408 PMC9896327

[ref81] Shield JM, Hide R, Harvey P, Vrbova H and Tulloch J (1987) Hookworm (*Necator americanus*) and *Strongyloides fuelleborni*-like prevalence and egg count with age in highlands fringe people of Papua New Guinea. *Papua and New Guinea Medical Journal* 30(1), 21–26. 3475865

[ref82] Shield JM and Kow F (2013) A comparative study of intestinal helminths in pre-school-age urban and rural children in Morobe Province, Papua New Guinea. *Papua New Guinea Medical Journal* 56(1/2), 14–31. 25423854

[ref83] Solórzano-García B, White-Day JM, Gómez-Contreras M, Cristóbal-Azkárate J, Osorio-Sarabia D and Rodríguez-Luna E (2017) Coprological survey of parasites of free-ranging jaguar (*Panthera onca*) and puma (*Puma concolor*) inhabiting 2 types of tropical forests in Mexico. *Revista Mexicana de Biodiversidad* 88(1), 146–153.

[ref84] Speare R (1986) *Studies on the Taxonomy of Strongyloides*. Townsville, Australia: Nematoda; Strongyloididae. James Cook University of North Queensland.

[ref85] Speare R and Tinsley D (1986) *Strongyloides felis*: An” old” worm rediscovered in Australian cats. *Australian Veterinary Practitioner* 16, 10–18.

[ref86] Speare R and Tinsley DJ (1987) Survey of cats for *Strongyloides felis*. *Australian Veterinary Journal* 64(6), 191–192. doi:10.1111/j.1751-0813.1987.tb09682.x 3632502

[ref87] Stroffolini G, Tamarozzi F, Fittipaldo A, Mazzi C, Le B, Vaz Nery S and Buonfrate D (2023) Impact of preventive chemotherapy on *Strongyloides stercoralis*: A systematic review and meta-analysis. *PLoS Neglected Tropical Diseases* 17(7), e0011473. 37428815 10.1371/journal.pntd.0011473 PMC10358935

[ref88] Susilowati S (1985) *Kejadian Infestasi Cacing Nematoda Dalam Saluran Pencernakan Kucing Di Wilayah Surabaya Utara*. Surabaya: Universias Airlangga.

[ref89] Thamsborg SM, Ketzis J, Horii Y and Matthews JB (2017) Strongyloides spp. infections of veterinary importance. *Parasitology* 144(3), 274–284. 27374886 10.1017/S0031182016001116

[ref90] Thanchomnang T, Intapan PM, Sanpool O, Rodpai R, Tourtip S, Yahom S, Kullawat J, Radomyos P, Thammasiri C and Maleewong W (2017) First molecular identification and genetic diversity of *Strongyloides stercoralis* and *Strongyloides fuelleborni* in human communities having contact with long-tailed macaques in Thailand. *Parasitology Research* 116(7), 1917–1923. 28500375 10.1007/s00436-017-5469-z

[ref91] Tobon Ramos JA, Maure T, Carias L, Lew D, Goss C, Samuel A, Tavul L, Fischer PU, Weil GJ and Laman M (2025) Impact of mass drug administration with ivermectin, diethylcarbamazine, and albendazole for lymphatic filariasis on hookworm and *Strongyloides stercoralis* infections in Papua New Guinea. *PLoS Neglected Tropical Diseases* 19(3), e0012851. 40063867 10.1371/journal.pntd.0012851 PMC11893124

[ref92] Unterköfler MS, Eipeldauer I, Merz S, Pantchev N, Hermann J, Brunthaler R, Basso W and Hinney B (2022) Strongyloides stercoralis infection in dogs in Austria: Two case reports. *Parasites and Vectors* 15(1), 168. 35570317 10.1186/s13071-022-05270-2 PMC9107779

[ref93] Vince JD, Ashford RW, Gratten MJ and Bana-Koiri J (2005) Stronglyloides species infestation in young infants of Papua New Guinea: Association with generalized oedema. *Papua New Guinea Medical Journal* 48(1/2), 50–57. 16894836

[ref94] Viney M and Ashford R (1990) The use of isoenzyme electrophoresis in the taxonomy of *Strongyloides*. *Annals of Tropical Medicine and Parasitology* 84(1), 35–47. 2331174 10.1080/00034983.1990.11812431

[ref95] Viney M, Ashford R and Barnish G (1991) A taxonomic study of *Strongyloides* Grassi, 1879 (Nematoda) with special reference to *Strongyloides fuelleborni* von Linstow, 1905 in man in Papua New Guinea and the description of a new subspecies. *Systematic Parasitology* 18(2), 95–109.

[ref96] Wallace FG, Mooney RD and Sanders A (1948) *Strongyloides fülleborni* infection in man. *American Journal of Tropical Medicine & Hygiene* 28(2), 299–302. doi:10.4269/ajtmh.1948.s1-28.299 18858031

[ref97] Wulcan JM, Dennis MM, Ketzis JK, Bevelock TJ and Verocai GG (2019) *Strongyloides* spp. in cats: A review of the literature and the first report of zoonotic *Strongyloides stercoralis* in colonic epithelial nodular hyperplasia in cats. *Parasites and Vectors* 12, 1–12. 31300009 10.1186/s13071-019-3592-7 PMC6626353

[ref98] Yamada M, Matsuda S, Nakazawa M, and Arizono N (1991) Species-specific differences in heterogonic development of serially transferred free-living generations of *Strongyloides planiceps* and *Strongyloides stercoralis*. *The Journal of Parasitology* 77, 592–594. 1865267

[ref99] Zhao H and Bradbury RS (2024) Feline strongyloidiasis: An insight into its global prevalence and transmission cycle. *One Health* 19, 100842. doi:10.1016/j.onehlt.2024.100842 39026543 PMC11255105

[ref100] Zhao H, Haidamak J, Noskova E, Ilik V, Pafčo B, Ford R, Masiria G, Maure T, Kotale N, Pomat W, Gordon C, Navarro S, Horwood PF, Constantinoiu C, Greenhill AR, and Bradbury RS (2025) New insights into infant strongyloidiasis in Papua New Guinea. *Emerging Infectious Diseases*. doi:10.3201/eid3109.241923. PMC12407202 40867023

